# Age-Related Changes in Cortical Connectivity During Surgical Anesthesia

**DOI:** 10.3389/fnagi.2019.00371

**Published:** 2020-01-10

**Authors:** Duan Li, Mike P. Puglia, Andrew P. Lapointe, Ka I Ip, Mackenzie Zierau, Amy McKinney, Phillip E. Vlisides

**Affiliations:** ^1^Department of Anesthesiology, University of Michigan Medical School, Ann Arbor, MI, United States; ^2^Center for Consciousness Science, University of Michigan Medical School, Ann Arbor, MI, United States; ^3^Department of Radiology, University of Calgary Cumming School of Medicine, Calgary, AB, Canada; ^4^Department of Psychology, University of Michigan, Ann Arbor, MI, United States

**Keywords:** aging, electroencephalography, intraoperative monitoring, neurophysiology, neurophysiological monitoring

## Abstract

An advanced understanding of the neurophysiologic changes that occur with aging may help improve care for older, vulnerable surgical patients. The objective of this study was to determine age-related changes in cortical connectivity patterns during surgical anesthesia. This was a substudy analysis of a prospective, observational study characterizing cortical connectivity during surgical anesthesia in adult patients (*n* = 45) *via* whole-scalp (16-channel) electroencephalography. Functional connectivity was estimated using a weighted phase lag index (wPLI), which was classified into a discrete set of states through k-means analysis. Temporal dynamics were quantified by occurrence rate and state transition probabilities. The mean global connectivity state transition probability [13.4% (±8.1)] was not correlated with age (*ρ* = 0.100, *p* = 0.513). Increasing age was inversely correlated with prefrontal-frontal alpha-beta connectivity (*ρ* = −0.446, *p* = 0.002) and positively correlated with frontal-parietal theta connectivity (*ρ* = 0.414, *p* = 0.005). After adjusting for anesthetic-related confounders, prefrontal-frontal alpha-beta connectivity remained significantly associated with age (*β* = −0.625, 95% CI −0.99 to −0.26; *p* = 0.001), while frontal-parietal theta connectivity was no longer significant (*β* = 0.436, 95% CI −0.03 to 0.90; *p* = 0.066). Specific transition states were also examined. Between frontal-parietal connectivity states, transitioning from theta-alpha to theta-dominated connectivity positively correlated with age (*ρ* = 0.545, *p* = 0.001). Dynamic connectivity states during surgical anesthesia, particularly involving alpha and theta bandwidths, maybe an informative measure to assess neurophysiologic changes that occur with aging.

## Introduction

Brain health is a growing concern among patients and perioperative physicians, particularly with aging surgical populations. In fact, the American Society of Anesthesiologists launched the Brain Health Initiative in 2015 (Fleisher, 2018), which aims to improve the understanding and management of postoperative neurocognitive recovery. A fundamental step for achieving this mission is to advance neurobiological understanding of the aging brain and related perioperative brain states, such as postoperative delirium. Identifying cortical biomarkers that correlate with age may be scientifically informative and clinically useful for predicting a propensity for deleterious perioperative brain states.

Studying cortical connectivity patterns during surgical anesthesia may serve as a useful method for identifying neurophysiological substrates of the aging brain. Electroencephalographic (EEG) analysis of oscillatory and connectivity patterns offers high temporal granularity of brain state alterations with direct comparisons across age groups in the setting of a major functional perturbation (i.e., general anesthesia). Furthermore, functional connectivity assessed *via* phase-based EEG analysis, provides a surrogate assessment of information transmission across the brain (Lee and Mashour, 2018). In the non-surgical setting, accumulating evidence suggests that aging, along with pathologic states associated with older age (e.g., Alzheimer’s disease), is associated with reductions in brain network flexibility (Hellyer et al., 2015; Córdova-Palomera et al., 2017; Naik et al., 2017; Alderson et al., 2018). One proposed theory of cognitive aging views the senescence process as a metastable system that serves to preserve function but predicts impaired switching between network states (Naik et al., 2017). These concepts may be reflected by reduced connectivity state transition likelihood in older patients. Additionally, connectivity states within certain bandwidths may be implicated. In particular, alpha and theta may be important for information transfer between the anterior and posterior brain regions (Hillebrand et al., 2016). As such, determining how perioperative connectivity patterns change with age, both globally and within certain bandwidths, might inform our understanding of neurocognitive function and vulnerability.

The objective of this study was to determine age-related changes in cortical connectivity patterns during surgical anesthesia. Given that structural and functional changes occur with aging that reduce dynamic flexibility of brain networks (Hellyer et al., 2015; Naik et al., 2017), this study tested the hypothesis that connectivity state transition probability would decrease with advancing age during the stable phase of surgical anesthesia. By estimating functional connectivity states through phase-based analysis and machine learning techniques, and analyzing temporal changes in these connectivity state occurrences, global transition properties can be successfully analyzed. Additionally, a secondary line of analysis was performed to examine regional spectral and spatial connectivity states and associated dynamics (e.g., occurrence rate, transition likelihood) over the age spectrum of participants. Particular focus was placed on alpha and theta prefrontal-frontal and frontal-parietal connectivity given the postulated roles these bandwidths and regions play in altered states of consciousness (Ku et al., 2011; Hillebrand et al., 2016; Koch et al., 2016; Flores et al., 2017), respectively.

## Materials and Methods

This is a secondary analysis of a prospective observational study that analyzed dynamic cortical connectivity patterns during surgical anesthesia (*n* = 53 participants; Vlisides et al., 2019). All study procedures took place at Michigan Medicine (Ann Arbor, MI, USA), and approval was obtained from the University of Michigan Medical School Institutional Review Board for this substudy analysis (HUM00164708, date of approval: 6/25/2019). Written informed consent was obtained from all participants under the parent observational study (HUM00113764; Vlisides et al., 2019) and recruitment took place from March 2017 to August 2017.

Study inclusion criteria included adult surgical patients (≥18 years of age) requiring general anesthesia for non-cardiac, non-intracranial neurologic, and non-major vascular surgery (i.e., operating above the inguinal ligament). Exclusion criteria included the following: emergency surgery, surgery involving the head and neck, patients known to have a difficult airway, non-English speaking, or enrolled in a conflicting research study.

### Perioperative and Anesthetic Procedures

The parent observational study was pragmatic in nature, with the intent of characterizing cortical connectivity patterns in a real-world surgical setting, irrespective of anesthetic maintenance regimen or surgical subtype (Vlisides et al., 2019). Thus, perioperative management proceeded as deemed appropriate by clinical teams; no research protocol was implemented other than EEG data collection procedures. All patients were induced with propofol, and maintenance regimens were mixed and involved either inhalational agents or propofol infusions (Vlisides et al., 2019). The specific surgical procedure itself was not analyzed in relation to study outcomes.

### EEG Data Acquisition and Analysis

Preoperatively, a wireless, whole-scalp 16-channel silver/silver-chloride EEG system (Mobile-72 system, Cognionics, San Diego, CA, USA) was placed after measuring head circumference, and signal quality was established *via* laptop computer. The corresponding 10–20 montage channel locations are available in the Supplementary Figure S1. Data were recorded at 500 samples per second, and impedances were maintained below 100 kΩ per manufacturer recommendations. The EEG recording software was synchronized with the electronic medical record in order to timestamp critical events (e.g., induction, intubation, skin incision), and a research assistant remained in the room for the entire case to accurately document the timing of such events. After study completion, raw EEG signals were exported to MATLAB (version 2017a; MathWorks Inc., Natick, MA, USA) and down-sampled to 250 Hz. Full acquisition details are available as previously reported (Vlisides et al., 2019).

For this substudy analysis, EEG data were only analyzed from the maintenance anesthesia phase (i.e., from 30 s after skin incision to the last minimum alveolar concentration value of 0.7 towards the end of the procedure). Specifically, EEG data were analyzed from prefrontal (Fp1, Fp2), frontal (F5, F6, Fz), and parietal (P5, P6, Pz) regions given their postulated role in consciousness and anesthetic-induced unconsciousness (Ku et al., 2011; Koch et al., 2016; Flores et al., 2017). Functional connectivity among brain regions was estimated using a weighted phase lag index (wPLI; Vinck et al., 2011). This is a measure of phase synchronization that accounts only for non-zero phase lag/lead relationships. In this context, wPLI is relatively robust to volume conduction and reference montage. Between two neurophysiologic signals, if one signal consistently leads (or lags) the other, the phases are considered locked, and wPLI approaches 1 depending on the consistency of phase relationships. Alternatively, if the relationship between two signals is random, without any consistent phase relationship, then the wPLI value will be 0. To ascertain wPLI data, EEG signals were divided into 30-s windows at 10-s step sizes, which were then divided into 2-s sub-windows with 50% overlapping. The multitaper method (Mitra and Bokil, 2007) was then used to estimate the cross-spectral density with time-bandwidth product = 2 and the number of tapers = 3. WPLI values were then estimated, as a function of frequency, using a custom-written function adapted from the Fieldtrip Toolbox (Oostenveld et al., 2011). For this analysis, frontal-parietal, and prefrontal–frontal wPLI were calculated in the bandwidth between 0.5–35 Hz at 0.5 Hz step. Surrogate data were generated *via* the trial-shuffling method to mitigate potential bias of wPLI; subsequently, wPLI was calculated and subtracted from the original value as the final estimation of functional connectivity. Full methodological details related to wPLI are available as previously reported (Vlisides et al., 2019).

Lastly, temporal variations of connectivity were assessed over the entire anesthetic maintenance period as previously described (Vlisides et al., 2019). In brief, connectivity patterns were obtained using principal component analysis and k-means clustering. First, using principal component analysis, the 140-dimensional vector was reduced to 5-dimensional feature, and these patterns were classified into five clusters using the k-means algorithm with squared Euclidean distance and 100 replications of the initial centroids. The number of clusters and number of retained components were determined using the stability index, which quantifies the reproducibility of clustering solutions for the studied dataset, the amount of variance explained by principal components, and the interpretability of the clustering results (Lange et al., 2004). The results produced five distinct connectivity states, in addition to burst suppression, with distinct spatial and spectral properties over the anesthetic maintenance period (Vlisides et al., 2019). Given the focus on prefrontal-frontal and frontal-parietal oscillatory dynamics, these are the connectivity states further analyzed in this manuscript.

The cluster analysis also allowed for characterization of cortical connectivity data over time. For each subject, we quantified the occurrence rate that is defined as the fraction of time spent in a given connectivity state, compared to all states during anesthetic maintenance, for a given participant. We then assumed the connectivity state time sequence to be a Markov chain (i.e., the state transition depends only on the current state) and computed the state transition probability for each pair of states. We also computed the global state transition probability, which is defined as the number of state transitions between distinct states divided by the total time a subject spent in the anesthetic maintenance period.

### Study Outcomes

The primary outcome of this substudy is global transition probability. Lower (higher) values suggest that cortical connectivity is less (more) likely to transition to a distinct state, or equivalently, more (less) likely to be persistent in the same state. Such a global measure may serve as a surrogate assessment of neurophysiologic metastability (Tognoli and Kelso, 2014; Hudson, 2017).

Multiple secondary outcomes were also examined. First, occurrence rates for prefrontal-frontal (S1, S2) and frontal-parietal dominant connectivity states (S4, S5) were calculated. This line of analysis may help to determine how spatial and spectral properties of cortical connectivity change with age. This is particularly relevant given prior evidence suggesting that frontal alpha power becomes reduced with age (Purdon et al., 2015a) and cognitive reserve (Giattino et al., 2017). A reduced occurrence rate of prefrontal-frontal alpha connectivity (i.e., S1 or S2) might, for example, suggest impaired functional dynamics, such as reduced anteriorization (Tinker et al., 1977; Giattino et al., 2017). Second, transition probabilities were calculated both within and between prefrontal-frontal and frontal-parietal states to produce a correlation matrix. The rationale for this correlation matrix is two-fold: (1) to determine whether transitioning from prefrontal-frontal to frontal-parietal states might be affected by age, which may suggest impaired functional dynamics in older patients; and (2) whether age also affects the tendency to remain within certain regional and spatial connectivity states. State 3 (S3) reflected a state of prefrontal-frontal connectivity in the delta bandwidth (Vlisides et al., 2019). Given the focus on alpha connectivity and related functional dynamics in this investigation, S3 was not analyzed as part of this manuscript.

### Statistical Analysis

Descriptive statistics were first analyzed, and the distribution of continuous data was assessed *via* the Shapiro–Wilk Test. Correlation analysis was conducted *via* Pearson correlation or Spearman’s rank-order correlation, as appropriate, based on the distribution of the data. For examining occurrence rates, four different correlation analyses (i.e., S1, S2, S4, and S5 with age) were simultaneously tested; thus, p-values underwent Bonferonni-correction for multiple comparisons resulting in an adjusted alpha level of 0.0125. For significant correlations, multivariable regression analysis was then performed to adjust for anesthetic depth and inhalational agent, as described in the “Results” section. Anesthetic depth was measured *via* age-adjusted minimum alveolar concentration (Lerou, 2004) and included in linear regression modeling as a covariate for adjustment. For these analyses, occurrence rates were dependent variables, and age was an independent variable. Multiple transition probabilities were analyzed in a correlation matrix as described, and the false discovery rate was controlled with the Benjamini–Hochberg procedure (Benjamini and Hochberg, 1995) with a false discovery rate set to 20%. All analyses were performed with IBM SPSS (version 25.0 for Windows; IBM Corp., Armonk, NY, USA).

## Results

Of the 97 patients initially screened for study inclusion, 65 were enrolled, and 12 (18.5%) were ultimately withdrawn. Fifty-three patients completed the study, and eight of these patients were further excluded from analysis due to poor EEG quality. Thus, data were available for analysis from 45 participants (Supplementary Figure S2). Baseline demographic and anesthetic details are presented in Table 1.

**Table 1:** Demographic, surgical, and anesthetic characteristics presented.

	Surgical patients (*n* = 45)
Age, year (IQR)	49 (37–73)
Male sex, *n* (%)	24 (53)
Race, *n* (%)	
White	40 (89)
Black	3 (6.7)
Other	2 (4.4)
Type of surgery, *n* (%)	
Urology	20 (44)
Orthopedic	10 (22)
Plastics	6 (13)
Surgical oncology	5 (11)
Neurosurgery	2 (4.4)
Minimally invasive surgery	2 (4.4)
Anesthetic maintenance regimen, *n* (%)	
Isoflurane-nitrous	14 (31)
Sevoflurane-nitrous	13 (29)
Propofol-nitrous	7 (16)
Sevoflurane	4 (8.9)
Isoflurane	3 (6.7)
Isoflurane-sevoflurane-nitrous	2 (4.4)
Propofol	1 (2.2)
Desflurane	1 (2.2)

**Figure 1:**
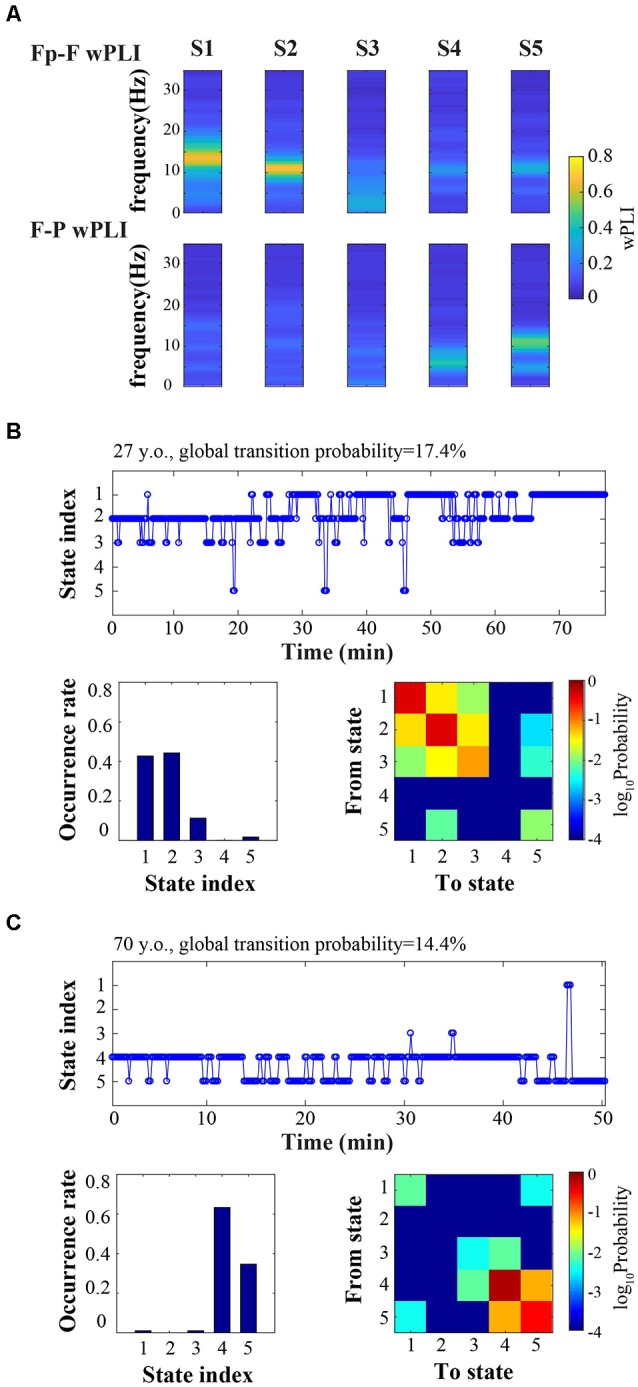
Cortical connectivity is dynamic during surgical anesthesia. **(A)** Representative connectivity states (S1, S2, S3, S4, S5) characterized by distinct frequency-resolved patterns of prefrontal-frontal (Fp-F) and frontal-parietal (F-P) weighted phase lag index (wPLI). **(B,C)** Time courses of connectivity states (top), occurrence rate (bottom left), and state transition matrix (bottom right) for an example younger subject (**B**, 27-year-old) and an example older subject (**C**, 70-year-old).

Connectivity analysis revealed distinct prefrontal-frontal and frontal-parietal states involving alpha and theta bandwidths (Figure 1A). Figure 1B provides an example of dynamic connectivity state fluctuation during surgical anesthesia in a young participant. Connectivity states predominantly reside in the prefrontal-frontal regions involving the alpha and beta bandwidths (S1 and S2). Conversely, Figure 1C provides an example of connectivity state transitions in an older patient during surgical anesthesia. Connectivity states primarily involve theta and alpha in the frontal-parietal regions.

### Global State Transitions

In terms of the primary outcome, the mean global transition probability was 13.4% (±8.1) among all participants. Overall transition probability was not correlated with age (*ρ* = 0.100, *p* = 0.513).

### Regional Connectivity Dynamics

#### Regional Connectivity Occurrence Rates

Connectivity state median occurrence rates and correlations with age are presented in Table 2. Prefrontal-frontal connectivity in the alpha and beta bandwidths (S1) was inversely correlated with age, while frontal-parietal theta connectivity (S4) was positively correlated with age (Table 2).

**Table 2:** Connectivity state occurrence rates and correlations with age.

Connectivity state	Occurrence rate, % (IQR)	Correlation with age (Spearman’s rho, *ρ*)	*P*-value
S1	3 (0–12)	−0.446	0.002^†^
S2	18 (2–56)	0.011	0.944
S4	9 (0–37)	0.414	0.005^†^
S5	7 (1–31)	0.235	0.121

*^†^Indicates statistical significance after Bonferonni correction. S1, prefrontal-frontal alpha-beta connectivity; S2, prefrontal-frontal alpha connectivity; S4, frontal-parietal theta connectivity; S5, frontal-parietal theta-alpha connectivity; IQR, interquartile range*.

Multivariable regression analysis was then undertaken to adjust for anesthetic-related confounders. After adjusting for anesthetic depth and nitrous use, given the effect of nitrous on beta oscillations (Purdon et al., 2015b), age remained significantly associated with the S1 occurrence rate (*β* = −0.625, 95% CI −0.99 to −0.26; *p* = 0.001). After adjusting for anesthetic depth (age-adjusted minimum alveolar concentration), the association between age and the S4 occurrence rate was no longer statistically significant (*β* = 0.436, 95% CI −0.03 to 0.90; *p* = 0.066).

#### Regional Connectivity State Transitions

**Figure 2:**
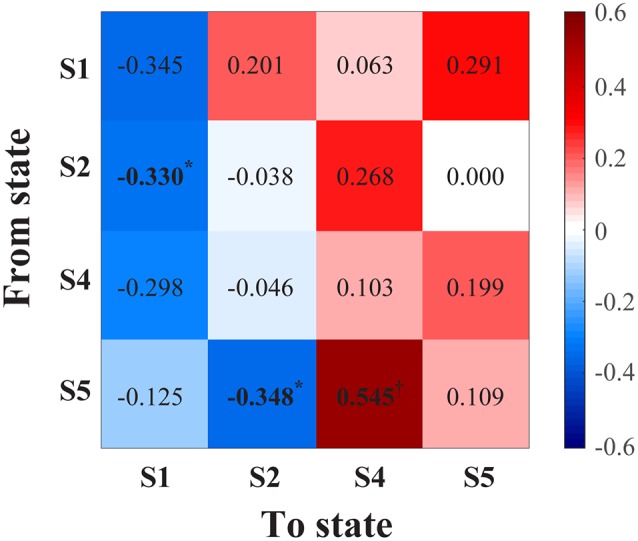
Correlation matrix (Spearman’s rho) between age and transition probability from a given connectivity state (y-axis) to another state (x-axis). *Indicates *P* < 0.05, but the values did not meet the critical threshold for significance *via* Benjamini–Hochberg criteria; ^†^indicates statistical significance (*P* = 0.001) meeting Benjamini–Hochberg threshold criteria. S1, prefrontal-frontal alpha-beta connectivity; S2, prefrontal-frontal alpha connectivity; S4, frontal-parietal theta connectivity; S5, frontal-parietal theta-alpha connectivity.

With increasing age, correlations were generally positive for transitions to frontal-parietal connectivity states (S4, S5), whereas transition probabilities to prefrontal-frontal connectivity states (S1, S2) were mostly negative (Figure 2). There was a statistically significant positive correlation with transitioning to frontal-parietal theta connectivity (S4) from the frontal-parietal alpha-theta connectivity state (S5; *ρ* = 0.545, *p* = 0.001; Figure 2). There were also inverse correlation trends between age and distinct transitions to prefrontal-frontal states (S2 to S1, *ρ* = −0.330, *p* = 0.037; S5 to S2, *ρ* = −0.348, *p* = 0.044), but these values resided above the Benjamini–Hochberg critical value for significance.

## Discussion

Studying dynamic transitions in functional connectivity is one possible avenue to advance understanding of the aging brain. In this study, we tested the hypothesis that the global connectivity state transition probability during the stable maintenance phase of surgical anesthesia would decrease with advanced age. Stated another way, as the brain ages, the propensity to undergo transitions between brain states may become less likely during general anesthesia. Contrary to our hypothesis, we found no correlation with the global transition probability and age; however, age was associated with the occurrence of specific regional and spectral brain states during surgical anesthesia. Specifically, younger patients tended to frequent states of prefrontal-frontal alpha-beta connectivity, and older patients tended to frequent frontal-parietal theta connectivity states.

Multiple reasons may account for the lack of an observed change in the global transition probability. First, the wPLI is an indirect, surrogate measure of functional connectivity. As a single measure, wPLI does not appear to distinguish levels of consciousness (Blain-Moraes et al., 2017; Vlisides et al., 2019) and may be unable to capture the rich repertoire of network changes that occur during anesthetic-induced altered states (Lee and Mashour, 2018). Advanced measures such as phase-lag entropy, partial phase locking, and network topology may be required to assess brain state transitions in a more refined manner (Lee et al., 2019). Another consideration is that stabilized brain states during anesthesia may have similar attractor properties despite different spectral or spatial characteristics (Hudson, 2017). That is, functional connectivity states may change with age based on region and oscillatory bandwidth, but global transition properties may remain similar. Thus, the qualitative nature of the transitions—rather than quantitative measures such as transition probability—may hold more relevance to the aging brain. Lack of correlation between age and global transition probability may have also been the result of anesthetic depth that was proportionally accounted for by age-based adjustment of anesthetic dosing. Indeed, there was no correlation between age and age-adjusted depth in this study, and changes in global transition probability may have been more pronounced if there was a larger discrepancy in anesthetic depth across participants. Overall, the mechanisms that drive brain state transitions during general anesthesia remain poorly understood, but the possibilities discussed offer testable hypotheses for future investigation.

Notably, two distinct prefrontal-frontal alpha connectivity patterns were observed in this analysis. Age was inversely correlated with the connectivity state (S1) that featured a broader range of frequencies (including beta) and higher peak frequency. These results parallel previous findings demonstrating a reduced peak alpha frequency in patients with age-related neuropathology (Moretti et al., 2004; Garcés et al., 2013). The broad connectivity range is also noteworthy. In a speculative context, this relatively wide range may reflect close proximity to network criticality. Criticality has been described as a state within a system that resides on the boundary between transitions (Lee et al., 2019), and functioning near criticality may confer neural network flexibility by enabling state transitions and promoting optimal information processing (Cocchi et al., 2017). Prefrontal-frontal alpha connectivity was one of the dominant patterns observed during general anesthesia, and the wide connectivity distribution around this peak in the alpha-beta connectivity state (S1) may reflect relative close proximity to criticality relative to the narrow alpha connectivity state (S2; Vlisides et al., 2019). Interestingly, the correlation matrix (Figure 2) suggested a reduced propensity to transition away from this state with advanced age. If this connectivity state resides in close proximity to criticality, this state may be more likely to occur with a younger age, which was observed in this study. However, these ideas reflect hypothesis-generating speculation and require further testing. Nonetheless, these distinct prefrontal-frontal connectivity states suggest a nuanced, complex milieu of connectivity dynamics that become fundamentally altered with aging. Alternatively, these results may be spurious given that there was no age-related correlation with the other prefrontal-frontal connectivity state (S2) and this was a secondary analysis. These findings should certainly be tested for replication, and further understanding, in subsequent investigations.

Theta frontal-parietal connectivity occurrence rate was initially correlated with age. However, after adjusting for anesthetic depth, this association was no longer statistically significant. Overall, results may nonetheless suggest a trend towards theta connectivity states with advancing age. Correlations were skewed positively between age and all transition pathways to frontal-parietal connectivity states involving theta (Figure 2), and this substudy analysis may have been underpowered for detecting the correlation strengths observed. These findings resonate with previous studies investigating theta, cognitive trajectory, and the aging brain. Theta power correlates with cognitive decline in older patients (Jelic et al., 2000; Prichep et al., 2006), and theta-to-alpha ratios correlate with cerebral hypoperfusion and concomitant neuropathology (e.g., Mild Cognitive Impairment, Alzheimer’s disease; Rodriguez et al., 1999; Mattia et al., 2003; Schmidt et al., 2013). Increases in age-related theta connectivity have also been demonstrated during working memory tasks (Hou et al., 2018). Although speculative, theta-to-alpha ratios may thus serve as a neurophysiologic marker of neurocognitive vulnerability during general anesthesia, similar to EEG suppression patterns (Fritz et al., 2018). Further investigation is certainly warranted with larger sample size, continued adjustment for anesthetic depth, ability to control for anesthetic effects on theta oscillations (Purdon et al., 2015b).

There are several limitations to consider when interpreting this study. First, this is an observational substudy that was not designed to address age-related differences in neurophysiology, and further investigation is required to support—or refute—these results. The parent study was a pragmatic design of patients undergoing surgical procedures, and surgical and anesthetic interventions were left to clinical discretion (Vlisides et al., 2019). Although connectivity states were consistent across anesthetic maintenance regimens, the possibility remains that specific volatile agents contribute to spectral patterns observed (Purdon et al., 2015b). These connectivity findings could be tested more rigorously with a single maintenance technique (e.g., propofol) in the future. Transitions among connectivity states represent unadjusted, bivariate correlations; additional biological factors that may confound transition patterns remain to be determined. Connectivity analysis was also limited to one measure (wPLI), and future studies should incorporate advanced measures of connectivity and network function. Neuroimaging (e.g., fMRI) could also be considered to provide further clarity with regards to structural and functional network alterations that become apparent with aging in the perioperative setting. Lastly, this study focused on adult patients at least 18 years of age. Future studies should investigate similar connectivity dynamics during younger developmental periods.

Dynamic connectivity states during the maintenance of general anesthesia, particularly involving the alpha and theta bandwidths, maybe an informative measure to assess neurophysiologic changes that occur with aging. In addition, these findings may serve to better inform the development of improved methods to monitor the aging brain during general anesthesia. Subsequent investigation is warranted to further understand the clinical and scientific relevance of these findings.

## Data Availability Statement

The datasets generated for this study are available on request to the corresponding author.

## Ethics Statement

This study was carried out in accordance with the recommendations of the University of Michigan Institutional Review Board. The protocol was also approved by the University of Michigan Institutional Review Board (HUM00113764, HUM00164708. Substudy approved on 6/25/2019). All subjects gave written informed consent in accordance with the Declaration of Helsinki.

## Author Contributions

DL, MP, and PV initially conceptualized the study idea and contributed to final data analysis. AL, KI, MZ, AM, and PV contributed to participant recruitment and data acquisition. All authors contributed to the final data interpretation. Lastly, all authors helped with manuscript preparation and revision for critically important content. The final version was approved by all authors, and each author agrees to be accountable for all aspects of the work.

## Conflict of Interest

The authors declare that the research was conducted in the absence of any commercial or financial relationships that could be construed as a potential conflict of interest.
