# Supplementary figures and images for: Age-Related Changes in Cortical Connectivity During Surgical Anesthesia
Source: Front Aging Neurosci. 2020 Jan 10;11:371. doi: 10.3389/fnagi.2019.00371 (PMC6967734; doi:10.3389/fnagi.2019.00371)

**Supplemental Digital Content**


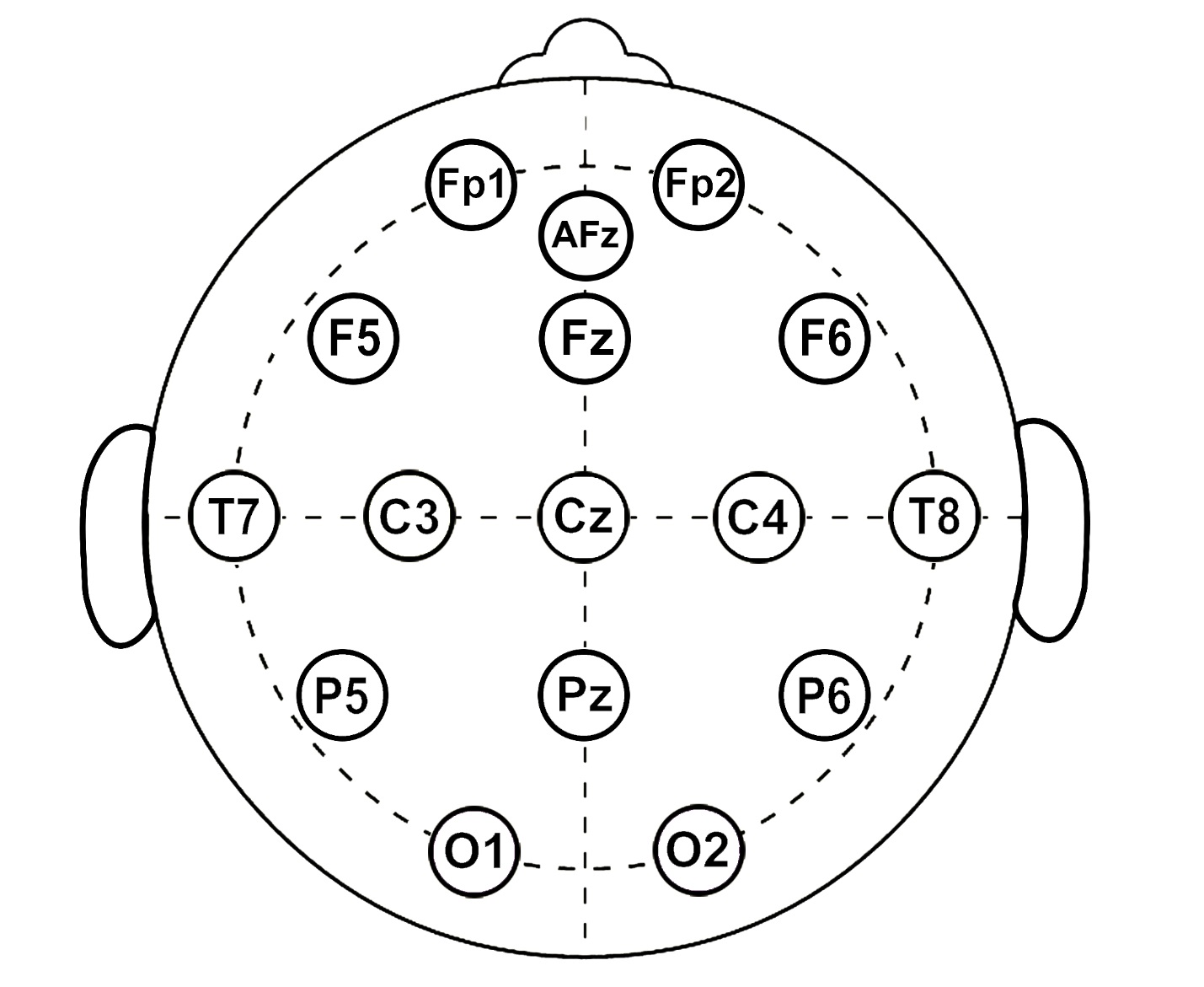


Figure 1: 16-Channel EEG Montage - International 10-20 System.

Supplement: Supplementary file 1 [file Table_1.DOCX]
