# Supplementary material for: Age-Related Changes in Cortical Connectivity During Surgical Anesthesia
Source: Front Aging Neurosci. 2020 Jan 10;11:371. doi: 10.3389/fnagi.2019.00371 (PMC6967734; doi:10.3389/fnagi.2019.00371)
Supplement: Supplementary file 2 [file Table_2.docx]

**Supplemental Digital Content**

97 Screened, eligible

Figure 2. Study flow diagram

45 Included in final analysis

8 Excluded

Poor EEG quality

12 Withdrawn

3 Anesthetic plan changed

2 Surgery rescheduled

2 Surgical plan changed - ineligible

2 Found to be medically ineligible

2 Participant withdrawal

1 Equipment failure

65 Enrolled

53 Completed study

32 Declined
